# Association of the Delaware Contraceptive Access Now Initiative with Postpartum LARC Use

**DOI:** 10.1007/s10995-022-03433-2

**Published:** 2022-04-30

**Authors:** Mónica L. Caudillo, Constanza Hurtado-Acuna, Michael S. Rendall, Michel Boudreaux

**Affiliations:** 1 Department of Sociology, University of Maryland, College Park, 3143 Parren Mitchell Art-Sociology Building, 3834 Campus Drive, College Park, MD 20742 USA; 2 Department of Health Policy and Management, University of Maryland, College Park, 4200 Valley Drive, Suite 2242, College Park, MD 20742 USA

**Keywords:** Long acting reversible contraceptives (LARCs), Medicaid, Postpartum contraceptive use, Provider training

## Abstract

**Objectives:**

Although multi-component policy interventions can be important tools to increase access to contraception, we know little about how they may change contraceptive use among postpartum women. We estimate the association of the Delaware Contraceptive Access Now (DelCAN) initiative with use of postpartum Long-Acting Reversible Contraception (LARC). DelCAN included Medicaid payment reform for immediate postpartum LARC use, provider training and technical assistance in LARC provision, and a public awareness campaign.

**Methods:**

We used a difference-in-differences design and data from the 2012 to 2017 pregnancy risk assessment monitoring system to compare changes in postpartum LARC use in Delaware versus 15 comparison states, and differences in such changes by women’s Medicaid enrollment.

**Results:**

Relative to the comparison states, postpartum LARC use in Delaware increased by 5.26 percentage points (95% CI 2.90–7.61, *P* < 0.001) during the 2015–2017 DelCAN implementation period. This increase was the largest among Medicaid-covered women, and grew over the first three implementation years. By the third year of the DelCAN initiative (2017), the relative increase in postpartum LARC use for Medicaid women exceeded that for non-Medicaid women by 7.24 percentage points (95% CI 0.12–14.37, *P* = 0.046).

**Conclusions for Practice:**

The DelCAN initiative was associated with increased LARC use among postpartum women in Delaware. During the first 3 years of the initiative, LARC use increased progressively and to a greater extent among Medicaid-enrolled women. Comprehensive initiatives that combine Medicaid payment reforms, provider training, free contraceptive services, and public awareness efforts may reduce unmet demand for highly effective contraceptives in the postpartum months.

**Supplementary Information:**

The online version contains supplementary material available at 10.1007/s10995-022-03433-2.

## Significance

*What is already known on this subject?* Postpartum women face multiple barriers to access highly effective contraception. Several states have implemented comprehensive family planning initiatives intended to reduce barriers to contraceptive use, but little is known about how they may change LARC use among postpartum women.

*What this study adds?* DelCAN, a statewide comprehensive family planning intervention, was associated with increased postpartum LARC use in Delaware, and this increase was the largest for women enrolled in Medicaid. States may improve access to effective contraceptives in the postpartum period by combining Medicaid payment reform, provider training, technical assistance, subsidies to outpatient contraceptive services, and public awareness campaigns.

## Introduction

Postpartum patients who wish to delay or prevent a subsequent pregnancy face unique difficulties to attain their reproductive goals, and have substantial unmet demand for highly effective contraception (Potter et al., 2014). Among other barriers, these patients often encounter bundled labor-and-delivery payments that do not separately reimburse for immediate postpartum (IPP) services and lack of widespread provider expertise with the most effective reversible contraceptives (Lacy et al., 2020; Okoroh et al., 2018; Potter et al., 2014). To address this problem, multiple states have implemented a policy change that facilitated Medicaid reimbursement of long-acting reversible contraceptives (LARCs) placed immediately postpartum (IPP), during the same hospital admission for delivery. In South Carolina, which implemented such payment reform in 2012, LARC insertions increased by 5.6 percentage points among Medicaid-enrolled women, and the probability of preterm births and low birth weight decreased after the policy change (Steenland et al., 2022). However, experiences with IPP payment reform have been mixed, with states like Wisconsin seeing a change of less than 0.2 percentage points in postpartum LARC use (Kramer et al., 2021). Furthermore, implementation of such reforms across healthcare facilities within participating states has been uneven (Okoroh et al., 2018; Steenland et al., 2021).

Given the wide variation in experiences with IPP payment reforms, it is key to identify circumstances under which these interventions can be successful. Previous studies have underscored the importance of providing training for healthcare providers and technical assistance to support the implementation of payment reforms (Fuerst et al., 2021; Lacy et al., 2020; Okoroh et al., 2018). And state-wide comprehensive family planning interventions encompassing information campaigns, no-cost contraceptive services, and/or provider training have been found to reduce barriers to contraceptive access among women of reproductive age (Biggs et al., 2015; Birgisson et al., 2015; Boudreaux et al., 2020; Lindo & Packham, 2017; Ricketts et al., 2014; Secura et al., 2014). Nonetheless, we know little about how such a comprehensive initiative may change contraceptive use among postpartum women.

This study contributes to the discussion on what works to reduce barriers to contraceptive access among postpartum women by assessing changes in their LARC use in Delaware relative to 15 comparison states after the implementation of the Delaware Contraceptive Access Now (DelCAN) initiative. DelCAN encompassed Medicaid payment reform to separately reimburse for IPP LARC devices, provision of free LARCs in outpatient clinics, provider training and technical assistance, and a public awareness campaign, all oriented towards increasing access to all contraceptive methods in general, and to LARCs in particular.

## Methods

### Intervention

The Delaware Contraceptive Access Now (DelCAN) initiative was launched in 2015 by Upstream USA and the state of Delaware, with the goal of ensuring same-day access to all contraceptive methods, and to LARCs in particular, regardless of a patient’s ability to pay. DelCAN ran from 2015 through early 2020 and encompassed several components focused on postpartum patients (Choi et al., 2020). In particular, Medicaid payment policy was altered to allow hospitals to obtain reimbursement for IPP LARC devices. Similar payment mandates were not issued for the privately insured. Hospitals obtained clinical and business operations training which was meant to address the billing and coding challenges that have been encountered in other states implementing IPP payment reform (Fuerst et al., 2021; Lacy et al., 2020). The largest delivery hospital in the state, responsible for approximately 50% of deliveries, had a strong champion provider and began offering IPP LARC services in 2015, while other hospitals joined on a rolling basis between 2016 and 2018.

In addition to hospital-based reforms, DelCAN also included outpatient reforms that may have increased access to contraceptives for postpartum patients. From February 2016 to March 2020, Upstream USA provided LARC training for medical providers and technical assistance to support staff in publicly funded Title X clinics, and in the largest private outpatient clinics in Delaware. Starting in 2015 and continuing through 2019, the Delaware Division of Public Health funded the acquisition of LARC devices, which enabled Title X clinics to provide these methods at no cost to women of any income level, therefore eliminating the sliding scale co-insurance fee that was previously in place for women above the poverty line. Finally, the public awareness campaign “Be Your Own Baby” was fielded from May 2017 to October 2018 to help patients find healthcare providers who could provide free or low cost same-day contraceptives.

To evaluate changes in postpartum LARC use after DelCAN, our analytical design offers three innovations relative to studies assessing similar outcomes after a payment policy change (Liberty et al., 2020; Smith et al., 2021; Steenland et al., 2021). First, we use survey-based, state-representative measurement that assesses LARC use among all postpartum women, not just LARC insertion rates among those enrolled in Medicaid. Second, we use comparison states to account for national trends in LARC use. Third, we assess heterogeneity in LARC use change by Medicaid enrollment. We offer details on each of these points below.

### Data Sources

We used data from the 2012 to 2017 Pregnancy Risk Assessment Monitoring System (PRAMS). The PRAMS is an annual, state-level probability survey representative of women who gave birth in each participating state and year. The PRAMS methodology and protocol have received approval from the Institutional Review Boards (IRB) of the Centers for Disease Control and Prevention and of participating states (Shulman et al., 2018). The University of Maryland IRB determined our analyses to be exempt from review. Our analysis was entirely based on survey and administrative data and did not use clinical study or patient data. Throughout the paper, we will sometimes refer to the DelCAN intervention as the “treatment” to adhere to the conventional language used in the difference-in-differences literature (Wing et al., 2018).

Our study period includes 3 years in the pre-treatment period (2012–2014), and 3 years in the treatment period (2015–2017). We included the 15 comparison states for which PRAMS data was available for all years in the study period: Alaska, Illinois, Massachusetts, Maryland, Maine, Missouri, New Jersey, New Mexico, Oklahoma, Pennsylvania, Utah, Washington, Wisconsin, West Virginia, and Wyoming. Colorado and South Carolina were excluded because they also implemented interventions to promote LARC use during the observed years (Liberty et al., 2020; Ricketts et al., 2014). We restricted our analytical sample to the 105,795 women who completed the survey between 2 and 9 months postpartum, who were neither pregnant nor trying to get pregnant at the time of the survey, and who were sexually active. We further excluded a weighted 7% who had missing values in any of the individual variables used in our models, for a final analytical sample of 93,285 women, with 4815 being from Delaware and 88,470 being from the 15 comparison states. The observation numbers reported above and in tables correspond to unweighted frequencies.

All PRAMS respondents were asked “Are you or your husband or partner doing anything *now* to keep from getting pregnant?,” and if they answered affirmatively, they were asked “What kind of birth control are you or your husband or partner using *now* to keep from getting pregnant?” Our outcome of interest is a binary variable (1 = yes, 0 = no) that identifies women who were using an implant or an IUD when they were surveyed. Respondents who were using any other contraceptive method, or no method at all, where coded in the reference category. Intervention status was measured by a binary variable that was equal to 1 if the respondent lived in Delaware, and 0 if she lived in any of the 15 comparison states.

The PRAMS asked women “During the *month before* you got pregnant with your new baby, what kind of health insurance did you have?” and “During your *most recent pregnancy*, what kind of health insurance did you have for your *prenatal care*?” Using the responses to these questions, we classified women as enrolled in Medicaid before or during pregnancy versus not enrolled. Women who become eligible for Medicaid during pregnancy have coverage for at least 60 days postpartum (Ranji et al., 2019). Those who were eligible for Medicaid coverage based on their pre-pregnancy income are likely to continue to be covered in the postpartum months.

Individual-level control variables from the PRAMS data included in our analysis were women’s age, race and ethnicity, and marital status, and characteristics of the recent birth, including pregnancy intention, birth order, birth weight, whether the birth was vaginal, and the infant’s age in months. State-level characteristics were added from other sources. We merged state-year data on the number of Federal Qualified Health Centers and rural health clinics per 100,000 women aged 15–50, obtained from the Health Resources & Services Administration (HRSA, 2020). We linked aggregate measures of sociodemographic conditions of women aged 15–50 for each state and year, obtained from the American Community Survey (Ruggles et al., 2019). These measures included the percentages of women living in poverty and with health insurance coverage. Finally, we merged Medicaid income eligibility thresholds for parents and pregnant women per state-year, obtained from the Kaiser Family Foundation (KFF, 2020). Medicaid eligibility rules helped to account for potential confounding from the Affordable Care Act’s Medicaid expansion.

### Statistical Analysis

We used difference-in-differences linear probability models (Wing et al., 2018) to estimate the associations of DelCAN with postpartum LARC use. The models compared changes in postpartum LARC use in Delaware, from before DelCAN to the first 3 years of DelCAN, to changes over the same period in the comparison states. The underlying assumption of the approach was that changes in the comparison group represent what would have happened in Delaware had DelCAN not been implemented. Because DelCAN was designed to influence LARC use through different mechanisms among women who were covered by Medicaid and women who were not, we estimated these models first for all women and then separately by Medicaid-coverage status.

All models included state and year fixed-effects, to which we added individual- and state-level time-varying controls. State fixed-effects account for sources of unobserved heterogeneity that may vary across states, but not over time, such as attitudes, cultural traits, or geographic characteristics. In contrast, year fixed effects capture unobserved confounders that vary over time, but are common to all states, such as macroeconomic trends.

Because only Medicaid-covered women were impacted by the IPP payment reform, and because those with and without Medicaid may have been affected by the outpatient components differently, we investigated if associations varied by Medicaid status. To do so, we pooled Medicaid and non-Medicaid women and included a triple interaction of Delaware, Medicaid status, and post-period indicators. This model also included individual controls, and Medicaid by state, Medicaid by year, and state by year interactions. We did not include state-level time-varying controls in these pooled models. In exploratory analyses, we estimated variations of these models in which we excluded state by year interactions and included state, year, and individual and state controls, all interacted by Medicaid, and obtained similar results.

We present results for two versions of each of the models described above. One in which time-relative to the intervention was measured as a binary pre-DelCAN to within-DelCAN (“pre-post”) variable, and one in which each year after the onset of DelCAN was allowed to have its own effect. The later of these approaches evaluated whether the effect of DelCAN was growing over time, coincident with increments in program components implemented over the 3 years (Choi et al., 2020).

Difference-in-differences models rely on the assumption that the trends in the comparison states accurately represent what would have occurred in Delaware had DelCAN not been implemented. Although this assumption cannot be directly tested, we follow the conventional approach (Ryan et al., 2015) and assess its plausibility by presenting a comparison of unadjusted trends in postpartum LARC use before and after the intervention started. All analyses were conducted in Stata 15. All our models adjusted for the complex survey design of PRAMS, using the Stata commands indicated in the survey documentation.

## Results

### Baseline Characteristics and Trends in LARC Use

Relative to women in the comparison states, in the baseline period women in Delaware were less educated, more likely to be enrolled in Medicaid before or during pregnancy, more likely to be Black, and less likely to be Hispanic (Table 1). Births to women in Delaware were also more likely to be unwanted or mistimed. See Online Appendix 2 for descriptive statistics of state-year variables.

**Table 1:** Sociodemographic characteristics of postpartum women^a^ in intervention and comparison states before DelCAN, 2012–2014

	All			Medicaid			No Medicaid		
	Delaware	Comparison states		Delaware	Comparison states		Delaware	Comparison states	
Sample size	2504	44,605		1210	21,069		1294	23,536	
Individual variables (%)									
Education			***			***			***
< HS	17.8 (16.3, 19.5)	12.5 (12.0, 12.9)		26.3 (23.7, 29.1)	21.6 (20.7, 22.5)		9.8 (8.2, 11.7)	6.0 (5.6, 6.5)	
High School	23.9 (22.2, 25.7)	23.3 (22.8, 23.9)		37.0 (34.1, 40.0)	37.1 (36.1, 38.1)		11.5 (9.8, 13.5)	13.7 (13.0, 14.3)	
Some College	29.5 (27.7, 31.4)	28.5 (27.8, 29.1)		30.7 (27.9, 33.5)	32.6 (31.6, 33.6)		28.5 (26.0, 31.1)	25.5 (24.8, 26.3)	
Bachelors+	28.7 (26.9, 30.6)	35.8 (35.1, 36.4)		6.0 (4.7, 7.6)	8.8 (8.2, 9.3)		50.2 (47.4, 53.1)	54.8 (53.9, 55.7)	
Age			***						
< 20	6.5 (5.5, 7.6)	5.7 (5.4, 6.1)		11.4 (9.6, 13.5)	11.0 (10.3, 11.7)		1.8 (1.2, 2.8)	2.1 (1.8, 2.4)	
20–24	22.8 (21.1, 24.6)	20.2 (19.6, 20.7)		33.1 (30.3, 36.0)	32.4 (31.4, 33.4)		13.1 (11.3, 15.2)	11.6 (11.0, 12.2)	
25–29	30.3 (28.4, 32.2)	30.5 (29.8, 31.1)		29.8 (27.1, 32.7)	30.1 (29.1, 31.1)		30.7 (28.1, 33.4)	30.7 (29.9, 31.5)	
30–34	26.6 (24.9, 28.4)	28.9 (28.3, 29.5)		17.6 (15.5, 20.0)	18.0 (17.2, 18.8)		35.1 (32.4, 37.9)	36.6 (35.7, 37.4)	
35+	13.8 (12.5, 15.3)	14.8 (14.3, 15.2)		8.1 (6.6, 9.9)	8.6 (8.0, 9.2)		19.3 (17.1, 21.6)	19.1 (18.4, 19.8)	
Race			***			***			***
Non-Hispanic White	56.5 (54.5, 58.6)	63.0 (62.4, 63.5)		45.3 (42.3, 48.4)	48.4 (47.5, 49.3)		67.2 (64.4, 69.8)	73.3 (72.6, 73.9)	
Non-Hispanic Black	24.2 (22.4, 26.0)	11.2 (10.7, 11.6)		35.4 (32.4, 38.4)	18.3 (17.5, 19.1)		13.6 (11.7, 15.7)	6.2 (5.8, 6.6)	
Non-Hispanic Asian	3.4 (2.8, 4.2)	5.0 (4.8, 5.2)		1.4 (0.8, 2.3)	3.1 (2.8, 3.4)		5.3 (4.2, 6.7)	6.4 (6.1, 6.7)	
Non-Hispanic, other	1.9 (1.4, 2.6)	4.3 (4.0, 4.5)		2.2 (1.5, 3.3)	5.9 (5.5, 6.3)		1.6 (1.0, 2.5)	3.1 (2.8, 3.4)	
Hispanic	14.0 (12.6, 15.5)	16.6 (16.2, 17.0)		15.7 (13.6, 18.0)	24.4 (23.7, 25.1)		12.4 (10.6, 14.4)	11.1 (10.7, 11.6)	
Married	52.0 (50.0, 54.1)	63.9 (63.2, 64.5)	***	24.4 (21.9, 27.0)	36.6 (35.6, 37.6)	***	78.3 (75.7, 80.6)	83.0 (82.3, 83.7)	***
Pregnancy intention index			***			***			***
Wanted sooner	9.9 (8.8, 11.2)	13.8 (13.3, 14.2)		5.2 (4.1, 6.7)	8.5 (7.9, 9.1)		14.3 (12.5, 16.4)	17.5 (16.8, 18.2)	
Unwanted or wanted later	36.1 (34.2, 38.2)	29.1 (28.4, 29.7)		48.9 (45.8, 52.0)	40.1 (39.1, 41.2)		24.1 (21.7, 26.6)	21.3 (20.5, 22.0)	
Right time or unsure	54.0 (51.9, 56.0)	57.2 (56.5, 57.9)		45.9 (42.9, 49.0)	51.4 (50.3, 52.5)		61.6 (58.8, 64.3)	61.3 (60.4, 62.1)	
Low birth weight	7.8 (7.7, 7.9)	6.7 (6.5, 6.9)	***	9.9 (9.7, 10.2)	7.8 (7.5, 8.1)	***	5.8 (5.7, 5.9)	5.9 (5.7, 6.2)	
Time since index birth			***			***			***
3 or 2 months	60.0 (58.0, 62.0)	35.5 (35.0, 36.1)		56.4 (53.3, 59.4)	29.6 (28.7, 30.5)		63.5 (60.7, 66.2)	39.7 (38.9, 40.5)	
4 to 6 months	39.0 (37.0, 41.0)	59.0 (58.3, 59.6)		42.5 (39.5, 45.6)	62.6 (61.6, 63.6)		35.7 (33.0, 38.5)	56.4 (55.6, 57.2)	
7 to 9 months	1.0 (0.6, 1.5)	5.5 (5.2, 5.8)		1.1 (0.6, 2.0)	7.8 (7.3, 8.4)		0.8 (0.4, 1.5)	3.9 (3.6, 4.2)	
Parity									
First birth	39.7 (37.7, 41.7)	39.1 (38.4, 39.8)		35.1 (32.2, 38.0)	34.7 (33.7, 35.7)		44.1 (41.3, 47.0)	42.2 (41.3, 43.1)	
2nd or 3rd	48.8 (46.8, 50.9)	50.1 (49.4, 50.8)		49.1 (46.1, 52.2)	50.4 (49.4, 51.5)		48.5 (45.7, 51.4)	49.9 (49.0, 50.8)	
4th or higher	11.5 (10.2, 12.8)	10.8 (10.4, 11.2)		15.8 (13.7, 18.1)	14.9 (14.1, 15.6)		7.4 (6.0, 9.0)	7.9 (7.5, 8.4)	
Vaginal birth	66.5 (64.6, 68.4)	68.2 (67.5, 68.8)		67.5 (64.6, 70.3)	69.0 (68.0, 70.0)		65.6 (62.9, 68.2)	67.6 (66.7, 68.4)	
Medicaid	48.7 (46.6, 50.7)	41.3 (40.7, 42.0)	***						

Data are % (95% CI) unless otherwise specified. Reported Ns are unweighted. Chi square tests were used to assess differences between Delaware and comparison states. Comparison states were AK, IL, MA, MD, ME, MO, NJ, NM, OK, PA, UT, WA, WI, WV, and WY

*Source:* 2012–2014 Pregnancy Risk Assessment Monitoring System (PRAMS)

^a^The analytical sample is restricted to postpartum women who were not pregnant or trying to get pregnant, and who were sexually active at the time of the survey

*P < 0.05, ** *P* < 0.01, *** *P* < 0.001

**Fig. 1:**
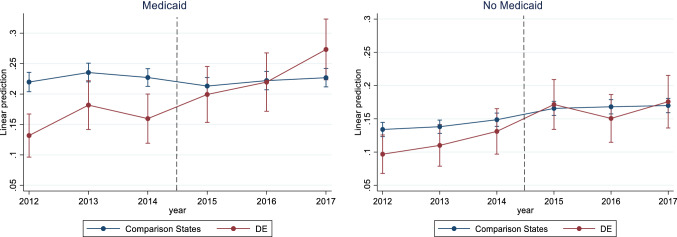
Trends in the probability of using LARCs postpartum by Medicaid-coverage status, 2012–2017. Comparison states in sample were AK, IL, MA, MD, ME, MO, NJ, NM, OK, PA, UT, WA, WI, WV, and WY. The analytical sample is restricted to postpartum women who were not pregnant or trying to get pregnant, and who were sexually active at the time of the survey. Vertical bar indicates the start of the Delaware Contraceptive Access Now (DelCAN) initiative in 2015. *Source:* Authors’ analyses of data from the 2012 to 2017 Pregnancy Risk Assessment Monitoring System (PRAMS)

Unadjusted trends show that postpartum LARC use increased for both Medicaid and non-Medicaid women for 2012–2017 in Delaware relative to the comparison states (Fig. 1). Increases in Delaware appear to be especially large for Medicaid-insured women, increasing from 16% in the pre-period to 27% in 2017. The trends in the probabilities of using LARCs postpartum during the study period appear parallel between treatment (Delaware) and control states prior to DelCAN (years 2012–2014). Statistical tests of the equality of pre-treatment unadjusted trends supported this observation: interactions between a linear pre-treatment time variable and Delaware versus comparison states were statistically non-significant for all, Medicaid, and non-Medicaid women (see Online Appendix 3).

### Difference-in-Differences Estimates

Results from our main models suggest that LARC use among postpartum women increased after DelCAN started, particularly among Medicaid-covered patients. In the adjusted difference-in-differences models using a binary pre-post treatment measure (Table 2), we found that after DelCAN came into effect the probability of postpartum LARC use increased by 5.26 percentage points among women in Delaware (95% CI 2.90–7.61) compared to women in control states. Postpartum LARC use increased in Delaware relative to the comparison states by 7.33 percentage points (95% CI 3.47–11.19) among Medicaid-covered women, and by 3.54 percentage points (95% CI 0.55–6.52) among those not covered by Medicaid. The difference in the changes in probability of LARC use between Medicaid- and non-Medicaid-covered women in the pooled model was 5.15 percentage points (95% CI 0.46–9.84).

**Table 2:** Difference-in-differences estimates for postpartum use of LARCs after DelCAN, and contrasts by Medicaid coverage status, 2012–2017

	% Using LARCs				Difference-in-differences estimate (95% CI)		*P*	(Medicaid—no Medicaid) contrast in difference-in-differences estimates (95% CI)		*P*
	2012–2014		2015–2017							
	DE	Other States	DE	Other States						
All women (N = 93,285)	13.5	17.6	19.6	18.9	5.26	(2.90–7.61)	< 0.001***	5.15	(0.46–9.84)	0.031*
Medicaid (N = 43,501)	15.8	22.7	23.1	22.1	7.33	(3.47–11.19)	< 0.001***			
No Medicaid (N = 49,784)	11.3	14.0	16.6	16.8	3.54	(0.55–6.52)	0.020*			

The analytical sample is restricted to postpartum women who were not pregnant or trying to get pregnant, and who were sexually active at the time of the survey

*DelCAN* Delaware contraception access now

Estimates are expressed in %. 95% CIs are in parentheses. Reported N’s are unweighted

Pre-DelCAN, 2012–2014; Post-DelCAN, 2015–2017

Comparison states in sample were AK, IL, MA, MD, ME, MO, NJ, NM, OK, PA, UT, WA, WI, WV, and WY

Difference-in-Differences = (Delaware_post-DelCAN_ − Delaware_pre-DelCAN_)  −  (Comparison_post-DelCAN_ − Comparison_pre-DelCAN_)

Difference-in-differences estimates were adjusted for women’s age, race/ethnicity, and marital status, and characteristics of the index birth, including pregnancy intention, birth order, birth weight, whether it was a vaginal birth, and the infant’s age in months. State-year controls included percentages of women living in poverty, and with health insurance coverage, clinics per 100,000 women aged 15–50, and Medicaid income thresholds for parents and pregnant women. Models included state and year fixed effects

Medicaid–no Medicaid contrast in difference-in-differences = ((Delaware_post-DelCAN_ − Delaware_pre-DelCAN_)  −  (Comparison_post-DelCAN_ − Comparison_pre-DelCAN_))_Medicaid_ −  ((Delaware_post-DelCAN_ − Delaware_pre-DelCAN_)  −  (Comparison_post-DelCAN_ − Comparison_pre-DelCAN_))_NoMedicaid_

The contrast in difference-in-differences estimates comes from a regression that included the individual controls listed above, and Medicaid by state, Medicaid by year, and state by year interactions

*Source:* Authors’ analyses of data from the 2012 to 2017 PRAMS

^*^P < 0.05, ** *P* < 0.01, *** *P* < 0.001

Models disaggregating post-treatment years suggest that after DelCAN started, the positive associations with the probability of postpartum LARC use in Delaware grew over time for all women and were driven by the Medicaid-enrolled (Table 3). For all postpartum women in Delaware, the increments in the probability of LARC use went from 4.67 percentage points in 2015 (95% CI 1.34–8.01), to 4.35 percentage points in 2016 (95% CI 0.94–7.75), and 7.21 percentage points in 2017 (95% CI 3.67–10.75). For Medicaid-enrolled women, the changes in postpartum LARC use went from increases of 5.58 and 6.77 percentage points in 2015 and 2016 to 10.5 percentage points (95% CI 4.75–16.27) in 2017, compared to Medicaid-covered women in comparison states. The increase in postpartum LARC use for Medicaid-covered women in 2017, the third year of the program, was statistically-significantly greater than that for women not covered by Medicaid by 7.24 percentage points (95% CI 0.12–14.37).

**Table 3:** Difference-in-differences estimates for postpartum use of LARCs comparing individual DelCAN years (2015–2017) to the pre-DelCAN period (2012–2014), and contrasts by Medicaid coverage status

	% Using LARCs				Difference-in-differences estimate (95% CI)		*P*	(Medicaid—not covered by Medicaid) contrast in difference-in-differences estimates (95% CI)		*P*
	2012–2014		2015							
	DE	Other states	DE	Other states						
2015										
All women (N = 16,068)	13.5	17.6	18.4	18.5	4.67	(1.34, 8.01)	0.006**	2.51	(− 4.25, 9.26)	0.467
Medicaid (N = 7472)	15.8	22.7	19.9	21.3	5.58	(0.21, 10.96)	0.042*			
No Medicaid (N = 8596)	11.3	14.0	17.2	16.6	3.77	(− 0.55, 8.09)	0.087			

2016										
All women (N = 15,180)	13.5	17.6	18.3	18.9	4.35	(0.94, 7.75)	0.012*	5.42	(− 1.42, 12.26)	0.120
Medicaid (N = 6914)	15.8	22.7	22.0	22.2	6.77	(1.16, 12.37)	0.018*			
No Medicaid (N = 8266)	11.3	14.0	15.1	16.8	2.34	(− 1.93, 6.61)	0.283			

2017										
All women (N = 14,928)	13.5	17.6	22.2	19.2	7.21	(3.67, 10.75)	< 0.001***	7.24	(0.12, 14.37)	0.046*
Medicaid (N = 6836)	15.8	22.7	27.3	22.7	10.51	(4.75, 16.27)	< 0.001***			
No Medicaid (N = 8092)	11.3	14.0	17.6	17.0	4.51	(0.01, 9.02)	0.050			

Estimates are expressed in %. 95% CIs are in parentheses. Reported N’s are unweighted

The analytical sample is restricted to postpartum women who were not pregnant or trying to get pregnant, and who were sexually active at the time of the survey

*DelCAN* Delaware contraception access now

Pre-DelCAN, 2012–2014; Post-DelCAN, 2015–2017

Comparison states in sample were AK, IL, MA, MD, ME, MO, NJ, NM, OK, PA, UT, WA, WI, WV, and WY

Difference-in-Differences = (Delaware_post-DelCAN_ − Delaware_pre-DelCAN_)  −  (Comparison_post-DelCAN_ − Comparison_pre-DelCAN_)

Difference-in-differences estimates were adjusted for women’s age, race/ethnicity, and marital status, and characteristics of the index birth, including pregnancy intention, birth order, birth weight, whether it was a vaginal birth, and the infant’s age in months. State-year controls included percentages of women living in poverty, and with health insurance coverage, clinics per 100,000 women aged 15–50, and Medicaid income thresholds for parents and pregnant women. Models included state and year fixed effects

Medicaid–no Medicaid contrast in difference-in-differences = ((Delaware_post-DelCAN_ − Delaware_pre-DelCAN_)  −  (Comparison_post-DelCAN_ − Comparison_pre-DelCAN_))_Medicaid_ −  ((Delaware_post-DelCAN_ − Delaware_pre-DelCAN_)  −  (Comparison_post-DelCAN_ − Comparison_pre-DelCAN_))_NoMedicaid_

The contrast in difference-in-differences estimates come from a regression that included the individual controls listed above, and Medicaid by state, Medicaid by year, and state by year interactions

*Source:* Authors’ analyses of data from the 2012 to 2017 PRAMS

*P < 0.05, ** *P* < 0.01, *** *P* < 0.001

### Sensitivity Tests

We replicated the models discussed above using two alternative comparison groups. First, one that excluded states that adopted ACA Medicaid expansion before 2017 (Online Appendix 4). Delaware covered parents and childless adults since prior to the ACA expansion and only moderately expanded eligibility as part of the ACA. Therefore, non-expansion states are similar to Delaware in that they did not experience as large of a change to Medicaid eligibility as was experienced by the typical expansion state. Second, using a comparison group that excluded states that implemented a reform intended to increase LARC use before 2017 (Online Appendix 5). As an additional sensitivity test, we restricted the Medicaid group to women who were enrolled before pregnancy, to exclude those who may have self-selected into Medicaid to obtain LARC under DelCAN (Online Appendix 6). Results from all these exercises are very similar to those in our original models.

As noted in the methods section, our primary statistical inference strategy was based on official PRAMS guidance which uses Taylor-series linearization to account for the complex sample design. However, difference-in-differences models are prone to serial autocorrelation at the level of the policy. Common strategies to account for this problem, including clustering, do not perform well in cases with only one treated unit in the treatment group and few clusters overall (Buchmueller et al., 2011; Ferman & Pinto, 2019). To address this concern, we conducted two sensitivity tests for the estimated p-values of our coefficients of interest. First, we used a bootstrap procedure to produce a set of p-values that properly accounts of autocorrelation with only one treated cluster (Ferman & Pinto, 2019) (Online Appendix 7). Second, we used permutation tests (Abadie et al., 2010; MacKinnon & Webb, 2020) (Online Appendix 8). In both sets of exercises, we found statistical inferences that were consistent with findings in our main analyses, which strengthens the evidence that our results are statistically meaningful.

## Discussion

The Delaware Contraceptive Access Now initiative combined multiple intervention components to address barriers to contraceptive access faced by postpartum women in Delaware. We find that DelCAN was associated with an increase in LARC use among postpartum women in the first 3 years of implementation. This result was robust to several sensitivity tests, and to alternative statistical inference strategies. The association between DelCAN and postpartum LARC use appears to be larger for Medicaid-covered women, consistent with their having received IPP LARC payment changes, an additional program component that was not available for non-Medicaid-enrolled women. However, the differential increments in LARC use by Medicaid status may also reflect heterogenous responses to the outpatient components that were designed to reach all reproductive age women in Delaware. Associations between DelCAN and postpartum LARC use also appear to have grown across the first 3 years of the DelCAN, which is consistent with the increase in the number of hospitals in Delaware providing IPP services and increased outpatient clinic capacity to perform LARC insertions over time (Choi et al., 2020).

Our results suggest that when combined with strong state government support, provider training, and technical assistance, initiatives that combine inpatient and outpatient components such as DelCAN may effectively expand access to highly effective contraception for postpartum women. The impact of DelCAN on postpartum LARC use may be at least comparable to that of successful similar initiatives. By the third year of DelCAN implementation, postpartum LARC use increased by 10.5 percentage points among Medicaid-enrolled women. This is equivalent to twice the increase in IPP LARC insertion among adult women 5 years after South Carolina’s IPP payment reform (Steenland et al., 2019). Differences in the magnitude of associations may be explained by the contribution of additional program components such as the media campaign, technical assistance, and provider training in DelCAN. Another potential explanation is that our study’s measurement allowed for both IPP and outpatient postpartum LARC insertions.

Our findings are particularly timely for policy makers and healthcare professionals given that as many as 43 states and the District of Columbia have reformed Medicaid payment for IPP LARC services in 2020 (ACOG, 2020), up from only 15 states out of 40 surveyed in 2014/2015 (Moniz et al., 2015). Furthermore, our results underscore the importance of postpartum Medicaid coverage, which the Medicaid and CHIP Payment and Access Commission recently recommended to extend to 12 months (Clark & Burak, 2021). Such recommendation was partially incorporated in the COVID-19 relief bill passed on March 11th 2021 (Associated Press, 2021).

Further insight about the role of public health interventions in reproductive autonomy can be gained from evaluating changes in use of alternative contraceptive methods. For instance, family planning initiatives may promote reproductive autonomy by allowing women to substitute sterilization with a LARC, a contraceptive strategy that offers similar effectiveness, while also being able to be reversed to adjust to their future fertility goals (Steenland et al., 2021). However, future research should assess the extent to which DelCAN expanded fully informed and free access to all types of contraceptive methods among postpartum women (Senderowicz, 2020).

Previous studies have documented biases in the healthcare system towards disproportionately limiting the fertility of disadvantaged patients (Manzer & Bell, 2021). Therefore, further research is also needed to ascertain if the increase in LARC use that we observed was produced by interactions centered around patient’s preferences (Dehlendorf et al., 2015), and if they were accompanied by unrestricted and timely access to LARC removal (Higgins et al., 2016). As family planning initiatives like DelCAN continue to be designed, implemented, and evaluated, especial attention should be paid to respecting and advancing the reproductive autonomy of racial-ethnic and socioeconomic groups that have historically suffered from restrictions to their fertility (Gomez et al., 2014).

Our research strategy incorporates several strengths and innovations relative to previous studies, including the estimation of changes in LARC use for all women, not only for those who were enrolled in Medicaid; assessing heterogeneous effects by Medicaid coverage; and using a comparison group of states to account for national trends in LARC use. A limitation of our study is that our data do not allow us to distinguish between LARC insertions that occurred during the delivery stay, and those that occurred at a later, follow-up postpartum visit. Instead, our estimates offer a measure of the total prevalence of statewide LARC use during the postpartum months, which accounts for both inpatient and outpatient insertions and early removals.

Based on our findings, multi-component state-wide initiatives like DelCAN may be viewed as a promising strategy to reduce unmet demand for highly effective contraception among postpartum women in other states. Researchers, clinicians, and policy makers should continue to examine and improve the design and implementation of comprehensive family planning initiatives and develop their full potential to advance reproductive autonomy.

## Supplementary Information

Below is the link to the electronic supplementary material.

Supplementary file1 (DOCX 84 kb)

## Data Availability

Researchers can apply to the CDC for access to the 2012–2017 Pregnancy Risk Assessment Monitoring System (PRAMS).
