# Supplementary material for: Association of the Delaware Contraceptive Access Now Initiative with Postpartum LARC Use
Source: Matern Child Health J. 2022 Apr 30;26(8):1657–66. doi: 10.1007/s10995-022-03433-2 (PMC9055365; doi:10.1007/s10995-022-03433-2)
Supplement: Supplementary file 1 — Supplementary file1 (DOCX 84 kb) [file 10995_2022_3433_MOESM1_ESM.docx]

**Association of the Delaware Contraceptive Access Now Initiative with Postpartum LARC Use**

**Supplementary File**

**Appendix 1. Trends in the probability of using LARCs postpartum, 2012-2017 PRAMS**

*Notes:* Comparison states in sample were AK, IL, MA, MD, ME, MO, NJ, NM, OK, PA, UT, WA, WI, WV, and WY. The analytical sample is restricted to postpartum women who were not pregnant or trying to get pregnant, and who were sexually active at the time of the survey. Vertical bar indicates the start of the Delaware Contraceptive Access Now (DelCAN) initiative in 2015.

*Source:* Authors’ analyses of data from the 2012-2017 Pregnancy Risk Assessment Monitoring System (PRAMS)

**Appendix 2. Descriptive statistics of state-year variables**

The following table presents the distribution of state-year variables included in our difference-in-difference models in the pre-treatment period.

**Table 1.** **Descriptive statistics of state-year variables in intervention and comparison states before DelCAN (2012-2014)**

|  | **Delaware** | **Comparison States** |  |
| --- | --- | --- | --- |
| **Sample** | 2,504 | 44,605 |  |
| **State-year variables (%)** |  |  |  |
| Health Insurance Coverage | 90.2 (90.2,90.2) | 87.8 (87.8,87.8) | <0.001*** |
| Poverty | 41.4 (41.4,41.4) | 42.9 (42.9,42.9) | <0.001*** |
| **State-year variables (mean)** |  |  |  |
| Number of clinics per 100,000 women ages 15-50 | 1.3 (1.3,1.3) | 4.0 (4.0,4.0) | <0.001*** |
| Medicaid Threshold Parents | 1.3 (1.3,1.3) | 1.1 (1.1,1.1) | <0.001*** |
| Medicaid Threshold Pregnant | 2.1 (2.1,2.1) | 2.1 (2.1,2.1) | <0.001*** |
|  |  |  |  |

**P* < 0.05, ** *P* < 0.01, *** *P* < 0.001.

*Notes:* Data are % (95% CI) unless otherwise specified. Chi-square and t-tests were used to assess differences between Delaware and comparison states. The denominators for these variables are restricted to women aged 15 – 50 in each state-year; the exception are Medicaid Thresholds. Medicaid Income Eligibility Limits for parents and pregnant women are defined as a ratio of the Federal Poverty Level. Sources: American Community Survey, Health Resources & Services Administration, and Kaiser Family Foundation.

**Appendix 3. Statistical tests of pre-treatment trends**

To test the hypothesis of parallel trends in LARC use in Delaware and Comparison States in the pre-treatment period, we estimated linear probability models on LARC use controlling by pre-DelCAN years (2012 to 2014) as a continuous variable, a binary variable that distinguishes between Delaware and the comparison states, and the interaction of these two variables.^1^ Table 1 shows the interaction coefficients for all women, women with Medicaid, and women without Medicaid. Given the p-values of these interactions, we are unable to reject the null hypothesis of parallel trends in LARC rates in Delaware versus the comparison states in the pre-DelCAN period.

**Table 1. Test of equality of pre-treatment unadjusted trends using linear probability models to predict postpartum LARC use, 2012-2014 PRAMS**

| **All** | | |  | **Medicaid** | | |  | **No Medicaid** | | |
| --- | --- | --- | --- | --- | --- | --- | --- | --- | --- | --- |
| **Delaware*Year** | | ***P*** |  | **Delaware*Year** | | ***P*** |  | **Delaware*Year** | | ***P*** |
| **(95% CI)** | |  |  | **(95% CI)** | |  |  | **(95% CI)** | |  |
|  |  |  |  |  |  |  |  |  |  |  |
| 1.00 | (-0.81, 2.81) | 0.292 |  | 1.01 | (-1.89, 3.91) | 0.495 |  | 0.99 | (-1.37, 3.34) | 0.412 |
|  |  |  |  |  |  |  |  |  |  |  |

*Notes:* Comparison states in sample were AK, IL, MA, MD, ME, MO, NJ, NM, OK, PA, UT, WA, WI, WV, and WY. DelCAN started in 2015. The analytical sample is restricted to postpartum women who were not pregnant or trying to get pregnant, and who were sexually active at the time of the survey.

**Appendix 4. Replication of analysis after excluding states with Medicaid expansions from the comparison group**

The models below replicate Tables 2 and 3 in the paper using a subset of states in the comparison group. This subset excludes states that had implemented Medicaid expansions by July 2017 (KFF, 2020).

**Table 1. Difference-in-Differences estimates for postpartum use of LARCs after DelCAN, and contrasts by Medicaid coverage status in states with no Medicaid expansion, 2012-2017**

|  | **% Using LARC in Delaware during the Pre-implementation Period** |  | **Difference-in-Difference Estimate**  **(95% CI)** | | ***P*** |  | **(Medicaid – No Medicaid) Contrast in Difference-in-Difference Estimates (95% CI)** | | ***P*** |
| --- | --- | --- | --- | --- | --- | --- | --- | --- | --- |
| All women | 13.5 |  | 6.02 | (3.00, 9.05) | <0.001*** |  | 5.09 | (-0.01, 10.19) | 0.050 |
|  |  |  |  |  |  |  |  |  |  |
| Medicaid | 15.8 |  | 7.28 | (3.13, 11.43) | 0.001** |  |  |  |  |
|  |  |  |  |  |  |  |  |  |  |
| No Medicaid | 11.3 |  | 5.01 | (1.48, 8.53) | 0.005** |  |  |  |  |
|  |  |  |  |  |  |  |  |  |  |

**P* < 0.05, ** *P* < 0.01, *** *P* < 0.001.

*Notes:* DelCAN, Delaware Contraception Access Now.

Data are % (95% CI). Pre-DelCAN, 2012-2014; Post-DelCAN, 2015-2017. All: N=40,770; Medicaid: N=19,525; No Medicaid: N=21,245. The analytical sample is restricted to postpartum women who were not pregnant or trying to get pregnant, and who were sexually active at the time of the survey.

Comparison states in sample were ME, MO, OK, UT, WI, and WY, which had not adopted Medicaid expansion by July 2017.

Difference-in-Difference = (Delaware_post-DelCAN_ - Delaware_pre-DelCAN_) - (Comparison_post-DelCAN_ - Comparison_pre-DelCAN_)

Difference-in-difference estimates were adjusted for women’s age, race/ethnicity, and marital status, and characteristics of the index birth, including pregnancy intention, birth order, birth weight, whether it was a vaginal birth, and the infant’s age in months. State-year controls included percentages of women living in poverty, and with health insurance coverage, clinics per 100,000 women aged 15-50, and Medicaid income thresholds for parents and pregnant women. Models included state and year fixed effects.

Medicaid–No Medicaid Contrast in Difference-in-Difference = ((Delaware_post-DelCAN_ - Delaware_pre-DelCAN_) - (Comparison_post-DelCAN_ - Comparison_pre-DelCAN_))_Medicaid_  -

((Delaware_post-DelCAN_ - Delaware_pre-DelCAN_) - (Comparison_post-DelCAN_ - Comparison_pre-DelCAN_))_NoMedicaid_

The contrast in difference-in-difference estimates comes from a pooled regression that included the individual controls listed above, and Medicaid by state, Medicaid by year, and state by year interactions.

*Source:* 2012-2014 Pregnancy Risk Assessment Monitoring System (PRAMS).

**Table 2. Difference-in-Differences estimates for postpartum use of LARCs after DelCAN comparing individual years post-treatment to the pre-period, and contrasts by Medicaid coverage status in states with no Medicaid expansion,** **2012-2017**

|  | **% Using LARC in Delaware during the Pre-implementation Period** | **2015**  **Difference-in-Difference Estimate (95% CI)** | | ***P*** |  | **(Medicaid – No Medicaid) Contrast in Difference-in-Difference Estimates (95% CI)** | | ***P*** |
| --- | --- | --- | --- | --- | --- | --- | --- | --- |
| All women | 13.5 | 5.53 | (1.77, 9.29) | 0.004** |  | 3.15 | (-4.20, 10.50) | 0.401 |
|  |  |  |  |  |  |  |  |  |
| Medicaid | 15.8 | 4.85 | (-0.61, 10.32) | 0.082 |  |  |  |  |
|  |  |  |  |  |  |  |  |  |
| No Medicaid | 11.3 | 5.79 | (1.18, 10.40) | 0.014* |  |  |  |  |
|  |  |  |  |  |  |  |  |  |
|  |  |  |  |  |  |  |  |  |
|  | **% Using LARC in Delaware during the Pre-implementation Period** | **2016**  **Difference-in-Difference Estimate (95% CI)** | | ***P*** |  | **(Medicaid – No Medicaid) Contrast in Difference-in-Difference Estimates (95% CI)** | | ***P*** |
| All women | 13.5 | 7.29 | (3.43, 11.15) | <0.001*** |  | 6.52 | (-0.87, 13.91) | 0.084 |
|  |  |  |  |  |  |  |  |  |
| Medicaid | 15.8 | 8.75 | (3.06, 14.44) | 0.003** |  |  |  |  |
|  |  |  |  |  |  |  |  |  |
| No Medicaid | 11.3 | 5.98 | (1.41, 10.55) | 0.010* |  |  |  |  |
|  |  |  |  |  |  |  |  |  |
|  |  |  |  |  |  |  |  |  |
|  | **% Using LARC in Delaware during the Pre-implementation Period** | **2017**  **Difference-in-Difference Estimate (95% CI)** | | ***P*** |  | **(Medicaid – No Medicaid) Contrast in Difference-in-Difference Estimates (95% CI)** | | ***P*** |
| All women | 13.5 | 7.89 | (3.75, 12.03) | <0.001*** |  | 5.27 | (-2.42, 12.97) | 0.179 |
|  |  |  |  |  |  |  |  |  |
| Medicaid | 15.8 | 10.45 | (4.52, 16.38) | 0.001** |  |  |  |  |
|  |  |  |  |  |  |  |  |  |
| Not Medicaid | 11.3 | 5.48 | (0.54, 10.41) | 0.030* |  |  |  |  |
|  |  |  |  |  |  |  |  |  |

**P* < 0.05, ** *P* < 0.01, *** *P* < 0.001.

*Notes:* DelCAN, Delaware Contraception Access Now.

Data are % (95% CI). Pre-DelCAN, 2012-2014; Post-DelCAN, 2015-2017. All: N=40,770; Medicaid: N=19,525; No Medicaid: N=21,245. The analytical sample is restricted to postpartum women who were not pregnant or trying to get pregnant, and who were sexually active at the time of the survey.

Comparison states in sample were ME, MO, OK, UT, WI, and WY, which had not adopted Medicaid expansion by July 2017.

Difference-in-Difference = (Delaware_post-DelCAN_ - Delaware_pre-DelCAN_) - (Comparison_post-DelCAN_ - Comparison_pre-DelCAN_)

Difference-in-difference estimates were adjusted for women’s age, race/ethnicity, and marital status, and characteristics of the index birth, including pregnancy intention, birth order, birth weight, whether it was a vaginal birth, and the infant’s age in months. State-year controls included percentages of women living in poverty, and with health insurance coverage, clinics per 100,000 women aged 15-50, and Medicaid income thresholds for parents and pregnant women. Models included state and year fixed effects.

Medicaid–No Medicaid Contrast in Difference-in-Difference = ((Delaware_post-DelCAN_ - Delaware_pre-DelCAN_) - (Comparison_post-DelCAN_ - Comparison_pre-DelCAN_))_Medicaid_  -

((Delaware_post-DelCAN_ - Delaware_pre-DelCAN_) - (Comparison_post-DelCAN_ - Comparison_pre-DelCAN_))_NoMedicaid_

The contrast in difference-in-difference estimates comes from a pooled regression that included the individual controls listed above, and Medicaid by state, Medicaid by year, and state by year interactions.

*Source:* 2012-2014 Pregnancy Risk Assessment Monitoring System (PRAMS).

**Appendix 5. Replication of analysis after excluding states that implemented a Medicaid-based IPP LARC reform from the comparison group**

The models below replicate Tables 2 and 3 in the paper using a subset of states in the comparison groups. This subset excludes states that had implemented a Medicaid-based IPP LARC reform before 2017 (ACOG, 2020; Moniz et al., 2015, 2016).

**Table 1. Difference-in-Difference estimates for postpartum use of LARCs after DelCAN, and contrasts by Medicaid coverage status in states with no Medicaid-based IPP LARC reforms, 2012-2017**

|  | **% Using LARC in Delaware during the**  **Pre-implementation Period** |  | **Difference-in-Difference Estimate (95% CI)** | | ***P*** |  | **(Medicaid – No Medicaid)**  **Contrast in Difference-in-Difference Estimates (95% CI)** | | ***P*** |
| --- | --- | --- | --- | --- | --- | --- | --- | --- | --- |
| All women | 13.5 |  | 5.51 | (2.98, 8.03) | <0.001*** |  | 4.17 | (-0.94, 9.28) | 0.110 |
|  |  |  |  |  |  |  |  |  |  |
| Medicaid | 15.8 |  | 7.79 | (3.57, 12.00) | <0.001*** |  |  |  |  |
|  |  |  |  |  |  |  |  |  |  |
| No Medicaid | 11.3 |  | 3.54 | (0.37, 6.72) | 0.029* |  |  |  |  |
|  |  |  |  |  |  |  |  |  |  |

**P* < 0.05, ** *P* < 0.01, *** *P* < 0.001.

*Notes:* DelCAN, Delaware Contraception Access Now.

Data are % (95% CI). Pre-DelCAN, 2012-2014; Post-DelCAN, 2015-2017. All: N=45,799; Medicaid: N=21,240; No Medicaid: N=24,559. The analytical sample is restricted to postpartum women who were not pregnant or trying to get pregnant, and who were sexually active at the time of the survey.

Comparison states in sample were AK, MA, ME, PA, UT, WI, WV, which had not adopted a Medicaid-based IPP LARC reform before 2017.

Difference-in-difference estimates were adjusted for women’s age, race/ethnicity, and marital status, and characteristics of the index birth, including pregnancy intention, birth order, birth weight, whether it was a vaginal birth, and the infant’s age in months. State-year controls included percentages of women living in poverty, and with health insurance coverage, clinics per 100,000 women aged 15-50, and Medicaid income thresholds for parents and pregnant women. Models included state and year fixed effects.

Medicaid–No Medicaid Contrast in Difference-in-Difference = ((Delaware_post-DelCAN_ - Delaware_pre-DelCAN_) - (Comparison_post-DelCAN_ - Comparison_pre-DelCAN_))_Medicaid_  -

((Delaware_post-DelCAN_ - Delaware_pre-DelCAN_) - (Comparison_post-DelCAN_ - Comparison_pre-DelCAN_))_NoMedicaid_

The contrast in difference-in-difference estimates comes from a pooled regression that included the individual controls listed above, and Medicaid by state, Medicaid by year, and state by year interactions.

*Source:* 2012-2014 Pregnancy Risk Assessment Monitoring System (PRAMS).

**Table 2. Difference-in-Difference estimates for postpartum use of LARCs after DelCAN comparing individual years post-treatment to the pre-period, and contrasts by Medicaid coverage status in states with no Medicaid-based IPP LARC reforms, 2012-2017**

|  | **% Using LARC in Delaware during the Pre-implementation Period** | **2015**  **Difference-in-Difference Estimate (95% CI)** | | ***P*** |  | **(Medicaid – No Medicaid) Contrast in Difference-in-Difference Estimates (95% CI)** | | ***P*** |
| --- | --- | --- | --- | --- | --- | --- | --- | --- |
| All women | 13.5 | 4.53 | (0.95, 8.11) | 0.013* |  | 3.22 | (-4.11, 10.55) | 0.390 |
|  |  |  |  |  |  |  |  |  |
| Medicaid | 15.8 | 6.77 | (1.20, 8.46) | 0.023* |  |  |  |  |
|  |  |  |  |  |  |  |  |  |
| No Medicaid | 11.3 | 2.77 | (3.26, 10.89) | 0.238 |  |  |  |  |
|  |  |  |  |  |  |  |  |  |
|  | **% Using LARC in Delaware during the Pre-implementation Period** | **2016**  **Difference-in-Difference Estimate (95% CI)** | | ***P*** |  | **(Medicaid – No Medicaid) Contrast in Difference-in-Difference Estimates (95% CI)** | | ***P*** |
| All women | 13.5 | 4.84 | (1.21, 8.46) | 0.009** |  | 3.51 | (-3.96, 10.99) | 0.357 |
|  |  |  |  |  |  |  |  |  |
| Medicaid | 15.8 | 6.05 | (-0.07, 12.17) | 0.053 |  |  |  |  |
|  |  |  |  |  |  |  |  |  |
| No Medicaid | 11.3 | 3.53 | (3.95, 16.56) | 0.123 |  |  |  |  |
|  |  |  |  |  |  |  |  |  |
|  | **% Using LARC in Delaware during the Pre-implementation Period** | **2017**  **Difference-in-Difference Estimate (95% CI)** | | ***P*** |  | **(Medicaid – No Medicaid) Contrast in Difference-in-Difference Estimates (95% CI)** | | ***P*** |
| All women | 13.5 | 7.16 | (3.34, 10.97) | <0.001*** |  | 5.48 | (-2.19, 13.14) | 0.161 |
|  |  |  |  |  |  |  |  |  |
| Medicaid | 15.8 | 10.26 | (-0.96, 8.03) | 0.001** |  |  |  |  |
|  |  |  |  |  |  |  |  |  |
| No Medicaid | 11.3 | 4.46 | (-0.37, 9.29) | 0.070 |  |  |  |  |
|  |  |  |  |  |  |  |  |  |

**P* < 0.05, ** *P* < 0.01, *** *P* < 0.001.

*Notes:* Data are % (95% CI). Pre-DelCAN, 2012-2014; Post-DelCAN, 2015-2017. All: N=45,799; Medicaid: N=21,240; No Medicaid: N=24,559. The analytical sample is restricted to postpartum women who were not pregnant or trying to get pregnant, and who were sexually active at the time of the survey.

Comparison states in sample were AK, MA, ME, PA, UT, WI, WV, which had not adopted a Medicaid-based IPP LARC reform before 2017.

Difference-in-Difference = (Delaware_post-DelCAN_ - Delaware_pre-DelCAN_) - (Comparison_post-DelCAN_ - Comparison_pre-DelCAN_)

Difference-in-difference estimates were adjusted for women’s age, race/ethnicity, and marital status, and characteristics of the index birth, including pregnancy intention, birth order, birth weight, whether it was a vaginal birth, and the infant’s age in months. State-year controls included percentages of women living in poverty, and with health insurance coverage, clinics per 100,000 women aged 15-50, and Medicaid income thresholds for parents and pregnant women. Models included state and year fixed effects.

Medicaid–No Medicaid Contrast in Difference-in-Difference = ((Delaware_post-DelCAN_ - Delaware_pre-DelCAN_) - (Comparison_post-DelCAN_ - Comparison_pre-DelCAN_))_Medicaid_  -

((Delaware_post-DelCAN_ - Delaware_pre-DelCAN_) - (Comparison_post-DelCAN_ - Comparison_pre-DelCAN_))_NoMedicaid_

The contrast in difference-in-difference estimates comes from a pooled regression that included the individual controls listed above, and Medicaid by state, Medicaid by year, and state by year interactions.

*Source:* 2012-2014 Pregnancy Risk Assessment Monitoring System (PRAMS).

**Appendix 6. Alternative Medicaid Enrollment Definition**

As a sensitivity test, we coded in the Medicaid category only those women who were enrolled strictly before pregnancy. We aimed to exclude any women who may have self-selected into this treatment group by seeking enrollment to benefit from DelCAN.

The models below replicate Tables 2 and 3 using this alternative Medicaid definition. Results are very similar and consistent with those in our main analysis.

**Table 1. Difference-in-Difference estimates for postpartum use of LARCs after DelCAN, and contrasts by Medicaid coverage status, 2012-2017**

|  | **% Using LARCs in Delaware during the Pre-implementation Period** |  | **Difference-in-Difference Estimate (95% CI)** | | ***P*** |  | **(Medicaid – No Medicaid) Contrast in Difference-in-Difference Estimates (95% CI)** | | ***P*** |
| --- | --- | --- | --- | --- | --- | --- | --- | --- | --- |
| All women (N=93,330) | 13.5 |  | 5.23 | (2.87, 7.59) | <0.001*** |  | 4.45 | (-0.71, 9.61) | 0.091 |
|  |  |  |  |  |  |  |  |  |  |
| Medicaid (N=23,658) | 14.3 |  | 7.46 | (2.85, 12.08) | 0.002** |  |  |  |  |
|  |  |  |  |  |  |  |  |  |  |
| No Medicaid (N=69,672) | 13.1 |  | 4.23 | (1.44, 7.02) | 0.003** |  |  |  |  |
|  |  |  |  |  |  |  |  |  |  |

**P* < 0.05, ** *P* < 0.01, *** *P* < 0.001.

*Notes:* Data are % (95% CI). Pre-DelCAN, 2012-2014; Post-DelCAN, 2015-2017. The analytical sample is restricted to postpartum women who were not pregnant or trying to get pregnant, and who were sexually active at the time of the survey.

Difference-in-Difference = (Delaware_post-DelCAN_ - Delaware_pre-DelCAN_) - (Comparison_post-DelCAN_ - Comparison_pre-DelCAN_)

Difference-in-difference estimates were adjusted for women’s age, race/ethnicity, and marital status, and characteristics of the index birth, including pregnancy intention, birth order, birth weight, whether it was a vaginal birth, and the infant’s age in months. State-year controls included percentages of women living in poverty, and with health insurance coverage, clinics per 100,000 women aged 15-50, and Medicaid income thresholds for parents and pregnant women. Models included state and year fixed effects.

Medicaid–No Medicaid Contrast in Difference-in-Difference = ((Delaware_post-DelCAN_ - Delaware_pre-DelCAN_) - (Comparison_post-DelCAN_ - Comparison_pre-DelCAN_))_Medicaid_  -

((Delaware_post-DelCAN_ - Delaware_pre-DelCAN_) - (Comparison_post-DelCAN_ - Comparison_pre-DelCAN_))_NoMedicaid_

The contrast in difference-in-difference estimates comes from a pooled regression that included the individual controls listed above, and Medicaid by state, Medicaid by year, and state by year interactions.

*Source:* 2012-2014 Pregnancy Risk Assessment Monitoring System (PRAMS).

**Table 2. Difference-in-Difference estimates for postpartum use of LARCs after DelCAN comparing individual years post-treatment to the pre-period, and contrasts by Medicaid coverage status, 2012-2017**

|  | **% Using LARC in Delaware during the Pre-implementation Period** |  | **2015**  **Difference-in-Difference Estimate (95% CI)** | | ***P*** |  | **(Medicaid – No Medicaid) Contrast in Difference-in-Difference Estimates (95% CI)** | | ***P*** |
| --- | --- | --- | --- | --- | --- | --- | --- | --- | --- |
| All women (N=93,330) | 13.5 |  | 4.61 | (1.28, 7.95) | 0.007** |  | 2.40 | (-4.85, 9.66) | 0.517 |
|  |  |  |  |  |  |  |  |  |  |
| Medicaid (N=23,658) | 14.3 |  | 5.56 | (-0.68, 11.80) | 0.081 |  |  |  |  |
|  |  |  |  |  |  |  |  |  |  |
| No Medicaid (N=69,672) | 13.1 |  | 4.37 | (0.34, 8.41) | 0.034* |  |  |  |  |
|  |  |  |  |  |  |  |  |  |  |
|  |  |  | **2016**  **Difference-in-Difference Estimate (95% CI)** | | ***P*** |  | **(Medicaid – No Medicaid) Contrast in Difference-in-Difference Estimates (95% CI)** | | ***P*** |
| All women | 13.5 |  | 4.35 | (0.94, 7.76) | 0.012* |  | 2.89 | (-4.59, 10.38) | 0.449 |
|  |  |  |  |  |  |  |  |  |  |
| Medicaid | 14.3 |  | 5.33 | (-1.30, 11.95) | 0.115 |  |  |  |  |
| No Medicaid |  |  | 3.98 | (-0.06, 8.01) | 0.054 |  |  |  |  |
|  |  |  |  |  |  |  |  |  |  |
|  |  |  | **2017**  **Difference-in-Difference Estimate (95% CI)** | | ***P*** |  | **(Medicaid – No Medicaid) Contrast in Difference-in-Difference Estimates (95% CI)** | | ***P*** |
| All women | 13.5 |  | 7.22 | (3.68, 10.76) | <0.001*** |  | 8.33 | (0.41, 16.25) | 0.039* |
|  |  |  |  |  |  |  |  |  |  |
| Medicaid | 14.3 |  | 12.4 | (5.35, 19.46) | 0.001** |  |  |  |  |
|  |  |  |  |  |  |  |  |  |  |
| No Medicaid | 13.1 |  | 4.59 | (0.45, 8.72) | 0.030* |  |  |  |  |
|  |  |  |  |  |  |  |  |  |  |

**P* < 0.05, ** *P* < 0.01, *** *P* < 0.001.

*Notes:* Data are % (95% CI). Pre-DelCAN, 2012-2014; Post-DelCAN, 2015-2017. The analytical sample is restricted to postpartum women who were not pregnant or trying to get pregnant, and who were sexually active at the time of the survey.

Difference-in-Difference = (Delaware_post-DelCAN_ - Delaware_pre-DelCAN_) - (Comparison_post-DelCAN_ - Comparison_pre-DelCAN_)

Difference-in-difference estimates were adjusted for women’s age, race/ethnicity, and marital status, and characteristics of the index birth, including pregnancy intention, birth order, birth weight, whether it was a vaginal birth, and the infant’s age in months. State-year controls included percentages of women living in poverty, and with health insurance coverage, clinics per 100,000 women aged 15-50, and Medicaid income thresholds for parents and pregnant women. Models included state and year fixed effects.

Medicaid–No Medicaid Contrast in Difference-in-Difference = ((Delaware_post-DelCAN_ - Delaware_pre-DelCAN_) - (Comparison_post-DelCAN_ - Comparison_pre-DelCAN_))_Medicaid_  -

((Delaware_post-DelCAN_ - Delaware_pre-DelCAN_) - (Comparison_post-DelCAN_ - Comparison_pre-DelCAN_))_NoMedicaid_

The contrast in difference-in-difference estimates comes from a pooled regression that included the individual controls listed above, and Medicaid by state, Medicaid by year, and state by year interactions.

*Source:* 2012-2014 Pregnancy Risk Assessment Monitoring System (PRAMS).

**Appendix 7. Alternative p-values using bootstrapping procedure**

We used a bootstrap procedure to produce a set of p-values that properly accounts of autocorrelation with only one treated cluster.^2^ Because this procedure is based on aggregating the data at the state-year level, the models used for bootstrapping include state-level controls but exclude individual-level controls to prevent aggregation bias. Nonetheless, point estimates and unadjusted p-values are virtually identical to those in Table 2 of the paper, even when individual-level controls are excluded. Bootstrapped p-values are larger than those in Table 2, but still significant at the 5% level for all and Medicaid-enrolled women.

**Table 1. Difference-in-Difference estimates for postpartum use of LARCs after DelCAN with regular and bootstrapped p-values, 2012-2017**

|  | | | | |
| --- | --- | --- | --- | --- |
|  | **Difference-in-Difference Estimate**  **(95% CI)** | | ***P*** | ***Bootstrapped P*** |
| All women (N=93,285) | 4.96 | (2.51-7.41) | <0.001*** | 0.018* |
|  |  |  |  |  |
| Medicaid (N=43,501) | 7.37 | (3.44-11.30) | <0.001*** | 0.049* |
|  |  |  |  |  |
| No Medicaid (N=49,784) | 3.38 | (0.32-6.44) | 0.030* | 0.067 |
|  |  |  |  |  |

**P* < 0.05, ** *P* < 0.01, *** *P* < 0.001.

*Notes:* DelCAN, Delaware Contraception Accesss Now.

Data are % (95% CI). Pre-DelCAN, 2012-2014; Post-DelCAN, 2015-2017. The analytical sample is restricted to postpartum women who were not pregnant or trying to get pregnant, and who were sexually active at the time of the survey.

Difference-in-Difference = (Delaware_post-DelCAN_ - Delaware_pre-DelCAN_) - (Comparison_post-DelCAN_ - Comparison_pre-DelCAN_)

Difference-in-differences estimates were adjusted for the following state-year controls: percentages of women living in poverty, and with health insurance coverage, clinics per 100,000 women aged 15-50, and Medicaid income thresholds for parents and pregnant women.

*Source:* 2012-2014 Pregnancy Risk Assessment Monitoring System (PRAMS).

**Appendix 8. Alternative p-values from permutation tests**

We used permutation tests based on the question of whether findings could be entirely explained by chance.^3^ To conduct these tests, we compared our estimates of the effect of DelCAN to 15 “placebo” estimates, obtained by switching the treatment group to each of the comparison states in 15 additional difference-in-difference exercises. We then used all of these values as the sampling distribution of our estimated treatment effect, and reported the p-value that corresponded to the percentile that the DelCAN effect represented in such distribution.^3,4^ Following convention, we considered *P*<.10 to be statistically significant in these permutation tests.^3^

The bootstrap procedure only allows us to estimate adjusted p-values for our main difference-in-difference models without the triple interactions, but we present p-values from permutation tests for all of our estimated treatment effects and Medicaid interactions. When using a binary pre-post treatment variable, permutation tests produced p-values below the conventional significance threshold for this type of procedure (*P*<.10) for treatment effects among all respondents, as well as among Medicaid and non-Medicaid respondents. We also obtained p-values lower than 0.10 for estimates using disaggregated post-treatment years for all respondents in years 2015, 2016, and 2017, and for Medicaid-covered women in 2017. Permutation tests produced a p-value below the 0.10 threshold for the contrast between the increases in postpartum LARC use for Medicaid and non-Medicaid women in 2017, which is consistent with findings in our main models.

Given that our sample includes only 16 states, the lowest possible permutation-based p-value is 0.06, and therefore our permutation tests are exceedingly conservative. Nonetheless, we obtained p-values below the .10 threshold for a large number of our estimates. These sensitivity tests strengthen the evidence that our results are statistically meaningful.

**Table 1. Difference-in-Difference estimates for postpartum use of LARCs after DelCAN, and contrasts by Medicaid coverage status, with p-values from permutation tests, 2012-2017**

|  | **Difference-in-Difference Estimate** | ***Permutation Test P*** |  | **(Medicaid – No Medicaid) Contrast in Difference-in-Difference Estimates** | ***Permutation Test P*** |
| --- | --- | --- | --- | --- | --- |
| All women (N=93,285) | 5.26 | 0.063 |  | 5.15 | 0.125 |
|  |  |  |  |  |  |
| Medicaid  (N=43,501) | 7.33 | 0.063 |  |  |  |
|  |  |  |  |  |  |
| No Medicaid (N=49,784) | 3.54 | 0.063 |  |  |  |
|  |  |  |  |  |  |

*Notes:* Data are % (95% CI).

DelCAN, Delaware Contraception Access Now. Pre-DelCAN, 2012-2014; Post-DelCAN, 2015-2017. The analytical sample is restricted to postpartum women who were not pregnant or trying to get pregnant, and who were sexually active at the time of the survey.

Difference-in-Difference = (Delaware_post-DelCAN_ - Delaware_pre-DelCAN_) - (Comparison_post-DelCAN_ - Comparison_pre-DelCAN_)

Difference-in-difference estimates were adjusted for women’s age, race/ethnicity, and marital status, and characteristics of the index birth, including pregnancy intention, birth order, birth weight, whether the baby was born through vaginal delivery, and the baby’s age in months. State-year controls included percentages of women living in poverty, and with health insurance coverage, clinics per 100,000 women aged 15-50, and Medicaid income thresholds for parents and pregnant women. Models included state and year fixed effects.

Medicaid–No Medicaid Contrast in Difference-in-Difference = ((Delaware_post-DelCAN_ - Delaware_pre-DelCAN_) - (Comparison_post-DelCAN_ - Comparison_pre-DelCAN_))_Medicaid_  -

((Delaware_post-DelCAN_ - Delaware_pre-DelCAN_) - (Comparison_post-DelCAN_ - Comparison_pre-DelCAN_))_NoMedicaid_

The contrast in difference-in-difference estimates comes from a pooled regression that included the individual controls listed above, and Medicaid by state, Medicaid by year, and state by year interactions.

*Source:* 2012-2014 Pregnancy Risk Assessment Monitoring System (PRAMS).

**Table 2. Difference-in-Difference estimates for postpartum use of LARCs after DelCAN comparing individual years post-treatment to the pre-period, and contrasts by Medicaid coverage status, with p-values from permutation tests, 2012-2017**

|  | **2015**  **Difference-in-Difference Estimate** | ***Permutation Test P*** |  | **(Medicaid – No Medicaid) Contrast in Difference-in-Difference Estimates** | ***Permutation Test P*** |
| --- | --- | --- | --- | --- | --- |
| All women (N=93,285) | 4.67 | 0.063 |  | 2.51 | 0.438 |
|  |  |  |  |  |  |
| Medicaid  (N=43,501) | 5.58 | 0.125 |  |  |  |
|  |  |  |  |  |  |
| No Medicaid (N=49,784) | 3.77 | 0.125 |  |  |  |
|  |  |  |  |  |  |
|  | **2016**  **Difference-in-Difference Estimate** | ***Permutation Test P*** |  | **(Medicaid – No Medicaid) Contrast in Difference-in-Difference Estimates** | ***Permutation Test P*** |
| All women | 4.35 | 0.063 |  | 5.42 | 0.125 |
|  |  |  |  |  |  |
| Medicaid | 6.77 | 0.125 |  |  |  |
|  |  |  |  |  |  |
| No Medicaid | 2.34 | 0.188 |  |  |  |
|  |  |  |  |  |  |
|  |  |  |  |  |  |
|  | **2017**  **Difference-in-Difference Estimate** | ***Permutation Test P*** |  | **(Medicaid – No Medicaid) Contrast in Difference-in-Difference Estimates** | ***Permutation Test P*** |
| All women | 7.21 | 0.063 |  | 7.24 | 0.063 |
|  |  |  |  |  |  |
| Medicaid | 10.51 | 0.063 |  |  |  |
|  |  |  |  |  |  |
| No Medicaid | 4.51 | 0.125 |  |  |  |
|  |  |  |  |  |  |

*Notes:* Data are % (95% CI).

DelCAN, Delaware Contraception Access Now. Pre-DelCAN, 2012-2014; Post-DelCAN, 2015-2017. The analytical sample is restricted to postpartum women who were not pregnant or trying to get pregnant, and who were sexually active at the time of the survey.

Difference-in-Difference = (Delaware_post-DelCAN_ - Delaware_pre-DelCAN_) - (Comparison_post-DelCAN_ - Comparison_pre-DelCAN_)

Difference-in-difference estimates were adjusted for women’s age, race/ethnicity, and marital status, and characteristics of the index birth, including pregnancy intention, birth order, birth weight, whether it was a vaginal birth, and the infant’s age in months. State-year controls included percentages of women living in poverty, and with health insurance coverage, clinics per 100,000 women aged 15-50, and Medicaid income thresholds for parents and pregnant women. Models included state and year fixed effects.

Medicaid–No Medicaid Contrast in Difference-in-Difference = ((Delaware_post-DelCAN_ - Delaware_pre-DelCAN_) - (Comparison_post-DelCAN_ - Comparison_pre-DelCAN_))_Medicaid_  -

((Delaware_post-DelCAN_ - Delaware_pre-DelCAN_) - (Comparison_post-DelCAN_ - Comparison_pre-DelCAN_))_NoMedicaid_

The contrast in difference-in-difference estimates comes from a pooled regression that included the individual controls listed above, and Medicaid by state, Medicaid by year, and state by year interactions.

*Source:* 2012-2014 Pregnancy Risk Assessment Monitoring System (PRAMS).

**References**

1. Zeldow B, Hatfield L. Difference-in-Difference. Accessed March 15, 2020. https://diff.healthpolicydatascience.org/

2. Ferman B, Pinto C. Inference in differences-in-differences with few treated groups and heteroskedasticity. *Review of Economics and Statistics*. 2019;101(3):452-467.

3. Abadie A, Diamond A, Hainmueller J. Synthetic control methods for comparative case studies: Estimating the effect of California’s tobacco control program. *Journal of the American Statistical Association*. 2010;105(490):493-505.

4. Buchmueller TC, DiNardo J, Valletta RG. The effect of an employer health insurance mandate on health insurance coverage and the demand for labor: Evidence from Hawaii. *American Economic Journal: Economic Policy*. 2011;3(4):25-51.
